# Differences in Clinical Manifestations of Human Parechovirus–Related Meningitis in Neonates After the COVID‐19 Pandemic: A Single‐Center Exploratory Study

**DOI:** 10.1155/ijpe/9170564

**Published:** 2026-02-12

**Authors:** Seong Wan Kim, Hyun Joo Jung, Yoong-A Suh, Jang Hoon Lee, Moon Sung Park, Seoheui Choi

**Affiliations:** ^1^ Department of Pediatrics, Ajou University School of Medicine, Suwon, Republic of Korea, ajou.ac.kr

**Keywords:** COVID-19, differences, encephalitis, meningitis, neonate, parechovirus

## Abstract

**Purpose:**

Human parechovirus (HPeV) is one of the major causes of viral meningitis in neonates. Following the COVID‐19 pandemic, a resurgence of HPeV infections was reported worldwide. This exploratory case series is aimed at describing the symptoms of neonatal HPeV meningitis occurring after the lifting of large‐scale quarantine measures.

**Methods:**

We retrospectively reviewed eight neonates admitted between June 1, 2023, and September 30, 2023, with fever and HPeV detected in cerebrospinal fluid (CSF) using the FilmArray Meningitis/Encephalitis Panel. Demographic variables, clinical presentation, laboratory data, respiratory support requirements, and treatment courses were analyzed.

**Results:**

Fever was the primary presenting symptom. Despite the absence of leukocytosis or pleocytosis, fever duration was prolonged (mean 4.3 days). Four patients (50%) exhibited respiratory symptoms requiring high‐flow nasal cannula support. Hyponatremia occurred in two neonates without neurological manifestations—an observation not commonly described in previous cohorts. No patients required invasive ventilation.

**Conclusion:**

This small, single‐center series suggests possible variations in neonatal HPeV presentation after the COVID‐19 pandemic; however, findings are preliminary and hypothesis‐generating. Larger multicenter studies with subtype analysis and long‐term neurodevelopmental follow‐up are essential.

## 1. Introduction

Human parechovirus (HPeV), a single‐stranded RNA virus belonging to the Picornaviridae family, is a well‐recognized cause of viral meningitis and sepsis‐like illness in neonates and young infants [1–7]. Clinical manifestations vary significantly depending on age and viral genotype, with HPeV‐3 reported as one of frequent causes of severe central nervous system (CNS) infection in neonates [6, 8–13]. Typical symptoms include fever, irritability, rash, poor feeding, gastrointestinal symptoms, and occasionally seizure; however, many infants exhibit normal cerebrospinal fluid (CSF) profiles without pleocytosis, often resulting in unnecessary antibiotic use [12, 14]. Because severe HPeV infection in neonates may be associated with long‐term neurodevelopmental impairment, early identification and careful monitoring remain essential [15].

The COVID‐19 pandemic brought unprecedented changes to global infectious disease epidemiology. Furthermore, during the COVID‐19 isolation period, substantial variations were observed in the incidence of infections among young children (e.g., Enterovirus) as well as in healthcare expenditures [16–20]. During 2020–2022, widespread implementation of masking, social distancing, and enhanced hygiene practices significantly reduced the circulation of many respiratory and enteric viruses. As quarantine measures were lifted and communities transitioned to postpandemic behaviors, several countries—including the United States, Australia, and parts of Asia—reported a resurgence of HPeV infections, particularly HPeV‐3 [12, 15, 21–23]. This unexpected uptick raised important clinical questions regarding whether neonatal presentations may differ from previously reported patterns. Despite these observations, there remains a lack of data describing postpandemic HPeV infections in neonates. In particular, it is uncertain whether clinical manifestations have shifted due to altered population‐level immunity, changes in viral epidemiology, or variations in neonatal susceptibility following reduced early‐life viral exposure during lockdown periods.

Therefore, this study sought not to determine definitive differences but rather to explore whether neonatal HPeV‐associated meningitis cases identified in 2023 showed clinical features that deviated from prepandemic descriptions. By characterizing the presentations of eight affected neonates in a single tertiary center following the COVID‐19 endemic transition, we aim to generate hypotheses that may guide future larger scale, multicenter investigations.

## 2. Material and Methods

### 2.1. Study Population

Among neonates less than 28 days of age or 44 weeks of corrected age or younger who visited Ajou University Hospital from June 1, 2023, to September 30, 2023, we investigated the clinical symptoms of eight patients with fever with HPeV detected in CSF.

### 2.2. Methods

A retrospective chart review was performed to determine each patient′s age, sex, gestational age, birth weight, clinical presentation, duration of symptoms, antibiotic use and duration, laboratory results, length of hospital stay, presence of siblings, and use and duration of respiratory support therapy. This study was designed as an exploratory retrospective case series including all available neonates diagnosed with HPeV‐related meningitis during the study period.

#### 2.2.1. Laboratory Test Materials

Peripheral blood test results (complete blood count [CBC], aspartate aminotransferase [AST], alanine transaminase [ALT], blood urea nitrogen [BUN], creatinine [Cr], glucose, sodium [Na], potassium [K], chlorine [Cl], calcium [Ca], C‐reactive protein [CRP], hydrogen ion concentration [pH], partial pressure of carbon dioxide [pCO_2_], bicarbonate [HCO_3_], and base excess [BE]), CSF test results (specific gravity [SG], white blood cell [WBC] count, red blood cell [RBC] count, protein, glucose, meningitis/encephalitis [ME] panel, and culture), and other infection screening test results (fecal rotavirus polymerase chain reaction [PCR] and respiratory PCR) were investigated. The use of respiratory support devices, the type of respiratory device, the duration of respiratory support therapy, and the use of other medications, including antibiotics, were recorded.

The fully automated multiplex PCR FilmArray Meningitis/Encephalitis (FA‐ME) Panel assay was used on CSF samples collected on admission to confirm HPeV infection (BioFire FilmArray Torch, BioFire Diagnostics, LLC, 515 Colorow Drive, Salt Lake City, UT 84108, United States). This microfilm array can detect six types of bacteria (*Escherichia coli* K1, *Haemophilus influenzae*, *Listeria monocytogenes*, *Neisseria meningitidis*, *Streptococcus agalactiae*, and *Streptococcus pneumonia*), seven types of viruses (Cytomegalovirus, Enterovirus, Herpes Simplex Virus 1, Herpes Simplex Virus 2, Human Herpesvirus 6, HPeV, and varicella‐zoster virus), and one fungus (*Cryptococcus neoformans*). In CSF examinations, pleocytosis was defined as more than 18 cells/high‐power field (HPF) in infants less than 28 days old.

### 2.3. Data Analysis

Descriptive statistics (rates, means, medians, and ranges) were calculated. The data were registered and analyzed using SPSS for Windows, Version 19.

### 2.4. Ethics Statement

Patient information was retrieved and evaluated with the approval of the Medical Records Department and the Institutional Review Board of Ajou University Hospital (IRB No. AJOUIRB‐DB‐2024‐014). The study was approved by the Regional Committee for Medical and Health Research Ethics.

## 3. Results

Regarding sex, a total of seven of the eight patients were girls (87.5%), the average gestational age at birth was 37.5 ± 2.8 weeks, the average weight at birth was 2940 ± 714 g, and the age at symptom onset was 23.3 ± 20.3 days after birth. One of these infants was a premature infant born at 30 + 2 weeks who became symptomatic at 34 + 4 weeks of corrected age, 37 days after discharge from the NICU. The main symptom was fever, the average maximum body temperature was 38.9^°^C ± 0.7^°^C, and hyperthermia lasted for an average of 4.3 ± 1.6 days. As with other accompanying symptoms, respiratory‐related symptoms such as apnea and desaturation were confirmed in four patients (50%). Although they required high‐flow nasal cannula (HFNC) therapy, these patients did not receive invasive ventilator therapy. The duration of HFNC therapy was 66.3 ± 23.3 h. The average length of hospitalization for the eight patients was 7.1 ± 2.0 days, and the average duration of antibiotic use was 3.4 ± 0.7 days. Antibiotic use was discontinued if the blood culture test performed at admission was negative for 72 h (Table 1).

**Table 1:** Demographic findings and clinical characteristics.

Patient	Sex	GA (weeks)	Bwt (g)	Age (days)	Environment	Highest temperature (°C)	Other combined symptoms
1	F	37 + 6	2940	28	Home (sibling)	38.9	
2	M	39 + 1	3340	13	Home (sibling)	38.6	Hyponatremia (115: 7.6 mEq/kg/day replace)
3	F	38 + 2	3500	11	Postpartum care center	38.2	Rotavirus coinfection, hyponatremia (126: 3.12 mEq/kg/day replace)
4	F	37 + 4	2740	18	Postpartum care center	38.4	
5	F	30 + 5 (CA 40 + 5)	1370	71	Home (sibling)	38.3	COVID‐19 coinfection
6	F	38 + 2	3170	22	Home (sibling)	38.8	
7	F	38 + 6	2800	14	Postpartum care center	40.2	
8	F	39 + 0	3660	9	Postpartum care center	39.4	
Average			2940 ± 714.1	23.3 ± 20.3		38.9 ± 0.7	

Abbreviations: Bwt, body weight; GA, gestational age.

According to the blood test, the average WBC count was 9812.5 ± 4673.8 (×10^3^), and the neutrophil ratio was 69.9*%* ± 11.8*%*. The average platelet count was 335.75 ± 52.2 k (×10^3^), and there were no patients with thrombocytopenia. The average CRP level was 0.48 ± 0.52 mg/dL, with mild elevation in two patients, and hyponatremia (Na < 135 mEq/L) was observed in two patients (25%), neither previously documented in neonatal HPeV meningitis cohorts. In one patient with hyponatremia, the Na level at the time of initial hospitalization was confirmed to be 124 mEq/L, and despite Na replacement, the patient′s level decreased to 115 mEq/L. The patient underwent Na replacement at a dosage of 7.6 mEq/kg/day, which improved after the fourth day of hospitalization. The other patient′s initial level was normal at 137 mEq/L, but due to apnea, HFNC therapy was applied, and the progression of hyponatremia was confirmed during follow‐up, with the level decreasing to 126 mEq/L. The patient improved on the second day after receiving 3.12 mEq/kg/day NaCl, and no neurological abnormalities, such as convulsions, were observed. Regarding the CSF test, HPeV was confirmed in all patients according to the CSF ME panel, the SG was 1.005 ± 0.0007, the WBC count was 0.75 ± 1.17(/*μ*L), the protein level was 54.8 ± 6.0 (mg/dL), the glucose level was 60.8 ± 13.1 (mg/dL), and pleocytosis was not confirmed (Table 2).

**Table 2:** Laboratory findings.

Patient	CBC WBC (×10^3^), Hb (g/dL)/Hct (%), PLT (×10^3^)	AST (U/L)/ALT (U/L)	CRP (mg/dL)	Electrolyte Na/K/Cl (mMol/L)	CSF WBC (/*μ*L)/RBC (/*μ*L)	CSF protein (mg/dL)/glucose (mg/dL)	Clinical symptoms	Respiratory support/duration (h)	Total duration of fever (days)/total duration of admission (days)
1	4.6 k, 9.1/26.9, 356 k	29/14	0.05	137/5.4/103	0/0	55.1/78	Fever, desaturation	HFNC, 41.9	4/5
2	11.4 k, 9.9/29.7, 353 k	29/10	0.35	124/4.8/92	0/0	52.7/54	Fever		7/10
3	13.5 k, 10.6/32.3, 330 k	19/11	0.44	137/5.3/105	0/0	52/76	Fever, desaturation	HFNC, 96.2	4/9
4	3.2 k, 14/41.8, 327 k	29/12	0.19	138/4.6/105	1/0	56.8/67	Fever, apnea, desaturation	HFNC, 71.7	4/6
5	6.1 k, 8.7/25.2, 417 k	39/21	1.09	136/4.1/104	0/0	64.1/66	Fever, desaturation	HFNC, 55.5	4/7
6	16.6 k, 11.2/33.8, 352 k	24/20	1.47	137/4.6/102	2/0	44.6/52	Fever		3/6
7	11.5 k, 13.3/40.2, 230 k	35/14	0.13	138/5.1/103	3/0	60.7/40	Fever		2/5
8	11.6 k, 14.2/40.7, 321 k	22/10	0.12	137/4.7/103	0/0	52/53	Fever		6/9
Average	9812.5 ± 4673.8 k, 11.4 ± 2.2/33.8 ± 6.5, 335.8 ± 52.2 k	28.3 ± 6.6/14.0 ± 4.3	0.48 ± 0.52	135.5 ± 4.7/4.8 ± 0.4/102.1 ± 4.2	0.8 ± 1.2/0	54.8 ± 6.0/60.8 ± 13.1			4.3 ± 1.6/7.1 ± 2.0

Abbreviations: ALT, alanine transaminase; AST, aspartate aminotransferase; CBC, common blood cell count; CRP, C‐reactive protein; CSF, cerebrospinal fluid; Hb, hemoglobin; Hct, hematocrit; HFNC, high‐flow nasal cannula; PLT, platelet; Prot, protein; WBC, white blood cell.

According to tests performed at the time of hospitalization to determine the cause of fever, one patient was confirmed to be coinfected with rotavirus, and one was coinfected with COVID‐19. Four patients had siblings, and the remaining four patients also had a history of receiving care at a postpartum care center.

## 4. Discussion

This exploratory case series describes the clinical characteristics of eight neonates diagnosed with HPeV meningitis in 2023, following the transition out of large‐scale COVID‐19 quarantine measures. While several findings were consistent with prior literature, a number of atypical or less commonly reported features were also observed. If fever is the only symptom of an infectious disease, problems such as excessive antibiotic use are likely to occur, so it is necessary to identify the exact cause of infection.

In this respect, it is important to know whether there are changes in the previously reported clinical manifestations of the disease [24]. First, when comparing the symptoms of neonates and young children with HPeV infection admitted to our hospital′s NICU in 2023, fever was the main symptom, but there were no symptoms such as rash, convulsions, diarrhea, or feeding difficulties, which are frequently reported in neonates. In particular, Yeom et al. [25] reported that a rash appears as fever disappears in Korean children, but in our sample, no patient experienced a rash. Second, although there are many reports that caution is needed for sepsis‐like symptoms such as hypotension [26], in our sample, blood pressure abnormalities, including hypotension, were not confirmed, and most of the other accompanying symptoms were respiratory‐related manifestations (50%). Desaturation was unrecognized by the caregivers and detected only on the monitoring after admission. The patients′ symptoms improved after HFNC therapy, a noninvasive assistive device, rather than with the use of an invasive ventilator. Third, according to a report by Cilla et al. [27], hyperthermia was reported to last approximately 1.25 days. A longer duration was observed in our sample (4.25 ± 1.59 days; Table 2), although this finding requires validation. The average length of hospitalization was reported to be approximately 9 days [2], but in our study, it was shorter, averaging 7.13 ± 1.96 days. Fourth, Muto et al. [28] reported a case of hyponatremia in a 1‐month‐old boy with hyperthermia and convulsions, but in our study, the two patients with hyperthermia did not have convulsions and were younger than 28 days of age. These cases occurred during the neonatal period, and symptoms were not previously reported.

Several previously published international cohorts that clearly specified an age of less than 30 days or corrected gestational age of less than 44 weeks and described clinical manifestations of HPeV infection were also reviewed. Notably, hyponatremia without neurological manifestations has rarely been documented in neonatal HPeV literature, and no similar findings were identified in the cohorts summarized in Table S1. Although the mechanism remains unclear, this observation warrants further investigation. Their key features are summarized in Table S1. Overall, our findings were generally consistent with previous reports; however, notable differences have also emerged. As summarized in Table S1, rash, seizure activity, and gastrointestinal symptoms were frequently reported in 30%–80% of neonatal HPeV cohorts prior to the pandemic, whereas none of the infants in our series exhibited rash or seizure, and gastrointestinal symptoms were uncommon. This pattern may suggest a shift in predominant symptom profiles following the COVID‐19 quarantine period. In other words, our findings were similar to those of previously published studies in that systemic symptoms such as fever were accompanied by respiratory symptoms, no leukocytosis indicated by blood and CSF test results, and the absence of increased inflammatory proteins in blood tests. However, in this study, neonatal symptoms such as a prolonged fever duration, an increased need for respiratory support, and hyponatremia without neurological symptoms such as convulsions occurred during the outbreak. Given that this study was a case series and did not include a comparison group, hypothesis testing or statistical comparison with historical norms could not be performed. To compensate for this limitation, we conducted a structured review of prior studies and summarized their key features in Table S1. While indirect, this approach provides contextual support for interpreting potential changes in symptomatology after the COVID‐19 pandemic.

This study has several limitations. First, we could not determine whether the observed symptom differences were due to virus pathogenicity or host response. Second, the small sample size without a power calculation limits statistical validity, and the findings should be regarded as exploratory. Third, the retrospective, single‐center design introduces bias and restricts generalizability. Fourth, HPeV subtyping was not performed, although previous studies in Korea have shown HPeV‐3 to be the predominant subtype in neonatal CNS infection [25, 29]; future work should incorporate genotyping to clarify subtype‐specific effects. Fifth, no neurodevelopmental follow‐up was conducted, despite the known long‐term risks associated with HPeV‐related CNS infection. Finally, it remains uncertain whether the increased prevalence of HPeV since 2022 reflects pandemic‐related shifts or simply the resurgence of primary infections following the lifting of quarantine measures. Because HPeV infections demonstrated a marked resurgence globally after 2022, these observations provide potentially useful insights into evolving patterns of neonatal disease, although causal conclusions cannot be drawn. Therefore, while causality cannot be inferred, the structured comparison with prior cohorts supports the possibility that postpandemic epidemiologic and immunologic shifts may have influenced clinical presentations.

However, despite these limitations, this study targeted a group of neonates/infants with delayed herd immunity to preexisting infectious diseases and studied changes in the clinical symptoms of preexisting infectious diseases that occurred after the end of quarantine, which was implemented to prevent the spread of new infectious diseases. If a new epidemic occurs in the future and quarantine is implemented, neonates/infants who are infected after quarantine should be carefully observed and treated, considering that the clinical symptoms of existing infectious diseases may differ after quarantine.

In summary, this study is hypothesis‐generating and offers preliminary evidence of postpandemic changes in HPeV‐related meningitis. Larger multicenter studies with long‐term neurodevelopmental follow‐up will be essential to validate and extend these findings. Taken together, these findings suggest potential alterations in neonatal HPeV manifestations in the postpandemic period; however, given the small sample size and lack of subtype analysis, these observations should be interpreted with caution. This study primarily serves to generate hypotheses and highlight emerging clinical patterns that require validation in larger, multicenter cohorts with genotyping and long‐term neurodevelopmental follow‐up.

## 5. Conclusions

In conclusion, while several atypical features—such as prolonged fever or hyponatremia—were observed, these findings remain preliminary due to the small sample size. This study provides hypothesis‐generating insights into possible alterations in neonatal HPeV manifestations after prolonged pandemic‐related quarantine. Future large‐scale studies with genotyping and long‐term neurodevelopmental follow‐up are essential.

## Funding

No funding was received for this manuscript.

## Ethics Statement

This study was performed in line with the principles of the Declaration of Helsinki. Approval was granted by the Medical Records Department and the Institutional Review Board of Ajou University Hospital (IRB No. AJOUIRB‐DB‐2024‐014).

## Conflicts of Interest

The authors declare no conflicts of interest.

## Supporting information


**Supporting Information** Additional supporting information can be found online in sthe Supporting Information section. File S1. Summary of previously published studies describing clinical manifestations of neonatal human parechovirus infection.

## Data Availability

The data that support the findings of this study are available from the corresponding author upon reasonable request.
