# Supplementary material for: Differences in Clinical Manifestations of Human Parechovirus–Related Meningitis in Neonates After the COVID‐19 Pandemic: A Single‐Center Exploratory Study
Source: Int J Pediatr. 2026 Feb 12;2026:9170564. doi: 10.1155/ijpe/9170564 (PMC12897580; doi:10.1155/ijpe/9170564)
Supplement: Supplementary file 1 — Supporting Information Additional supporting information can be found online in sthe Supporting Information section. File S1. Summary of previously published studies describing clinical manifestations of neonatal human parechovirus infection. [file IJPE-2026-9170564-s001.docx]

**Supplement 1**. Summary of previously published studies describing clinical manifestations of neonatal human Parechovirus infection.

| First Author | Age (days) | Main Clinical Manifestations | Lab findings |
| --- | --- | --- | --- |
| Boivin et al. (29) | 27 | Fever, no respiratory or gastrointestinal symptoms  After 1day tachypnea with desaturation, rash  3days later, symptoms improved except rash, HAD (5) | Culture negative  No Leukocytosis |
|  | 20 | Fever, with respiratory symptoms and rash, conjunctivitis  After 1day, desaturation with O2 therapy, AOM  3days later fever subsided, HAD (5) |  |
|  | 7 | Fever, irritability, poor feeding without respiratory/gastrointestinal infection, rash  After 1day, rash spread, AOM, tachypnea  Over 4days, improved fever, feeding, tachypnea, HAD (5) |  |
| Benschop et al. (30) | 6 | Fever, poor feeding  No respiratory / gastrointestinal problems |  |
| Verboon-Maciolek et al. (31) | < CA 44+0wks  (n=10) | Fever, Hypertonia, seizure, apnea, rash, irritability, diarrhea, lethargy | CSF involve 7cases |
| Gupta et al. (32) | 7 | Pyrexia, poor feeding, lethargy, seizure, Ventilator care | B-MRI: evolusion of the extensive symmetrical signal abnormalities into strikingly cystic white matter change |
| Yuzurihara et al. (33) | 14 | Fever(3), rash(+), abdominal distension(+), respiratory(-) | Negative Lab findings |
|  | 21 | Fever(3), Somnolence(+), Rash(+), Abdominal distenision(-) |  |
|  | 28 | Fever(5), Somnolence(+), Rash(-), Abdominal distension(-) |  |
| Piralla et al. (34) | <30 | Fever, irritability, hyporeactivity, hypotonia, vomiting, drowsiness, rash |  |
| Pariani et al. (35) | 16 | Hyporeactivity, reduced oral intake, fever, tachypnea, rash, abdominal distension, irritability, generalized hypertonia, seizure | B-USG: mild lenticulostriate vasculopathy  B-MRI: multiple small focal noncystic areas of increased signal on T2 weighted spin-echo sequences |
|  | 9 | Plaintive tears, reduced oral intake and fever, semiliquid stools, irritability, seizure | B-MRI: punctate white matter lesions(T2), diffuse excessive high signal intensity(Diffusion) |
| Hara et al. (36) | 8 | Fever(2), rash(-), diarrhea, oxygen(-), Apnea(-), HAD(7) |  |
|  | 18 | Fever(2), Rash(+), diarrhea(-), oxygen(+), Apnea(+), HAD(5) |  |
|  | 23 | Fever(3), rash(-), diarrhea(+), oxygen(+), apnea(+), HAD(6) |  |
| Skram et al. (1) | 12 | Fever(+), dehydration(-), mottling(+), abdominal distension(+), HAD(2) | Meningoencephalitis  Sepsis-like syndrome |
|  | 29 | Fever(+), Dehydration(-), mottling(+), Abdominal distension(-), HAD(5) | Neutropenia, Hepatitis |
|  | 9 | Fever(+), Dehydration(+), skin(-), abdominal distension(-), HAD(7) | Meningoencephalitis, ARI, Seizure, Sepsis-like syndrome, Pneumonia |
|  | 5 | Fever(+), Dehydration(+), mottling(+), Severe anal prolapse(+), HAD(4) | Meningoencephalitis, Sepsis-like syndrome |
|  | 24 | Fever(+), Dehydration(+), Mottling, Abdominal distension(+), HAD(8) | Meningoencephalitis, ARI, Seizure, sepsis-like syndrome, neutropenia, hepatitis, pneumonia, transient hypothyroidism |
|  | 11 | Fever(+), dehydration(-), skin(-), abdominal distension(-), HAD(3) | Sepsis-like syndrome, Bronchiolitis |
|  | 11 | Fever(+), Dehydration(+), Mottling(+), abdominal distension(+) | Meningoencephalitis, Sepsis-like syndrome |
|  | 14 | Fever(+), dehydration(-), mottling(+), abdominal distension(-), HAD(5) | Meningoencephalitis, Urinary tract infection |
|  | 14 | Fever(+), dehydration(+), rash(+), mottling(+), abdominal distension(+), HAD(34) | Meningoencephalitis, ARI, Seizure |
|  | 10 | Fever(+), dehydration(+), mottling(+), abdominal distension, HAD(8) | Meningoencephalitis, Sepsis-like syndrome |
|  | 25 | Fever(+), dehydration(+), rash(+), mottling(+), abdominal distension(+) | Sepsis-like syndrome |
| Cilla et al. (27) | 19 | Fever(+, 1.5)), vomits(+), diarrhea(+), apnea(+), irritability |  |
|  | 6 | Fever(+,1) |  |
| Brownell et al. (37) | 8 | Fussiness, difficulty feeding, grunting, inconsolable, increased tone, jaundice, rash, hypothermia, tachycardia, seizure, respiratory distress | B-MRI: significant white matter abnormalities |
|  | 6 | seizure | B-MRI: restricted diffusion extending from the periventricular white matter to the subcortical regions of both cerebral hemispheres |
| Leow et al. (38) | 9 | Irritability, poor feeding, apyrexial, deteriorated with apneas, encephalopathy needing ventilation | B-MRI: HPeV infection |
|  | 12 | Seizure, pyrexia |  |
| Britton et al. (39) | 9 | Lethargy, seizure, fever, irritability, poor feeding, rash, septic, HAD(13) |  |
|  | 12 | Lethargy, fever, irritability, rash, diarrhea, poor feeding, septic, HAD(12) |  |
|  | 8 | Lethargy, seizure, fever, poor feeding, rash, HAD(4) |  |
|  | 13 | Lethargy, decreased LOC, seizure, irritability, rash, HAD(7) |  |
|  | 10 | Lethargy, seizures, fever, irritability, poor feeding, rash, HAD(10) |  |
|  | 8 | Lethargy, fever, irritability, rash, hepatitis, cytopenias, HAD(6) |  |
|  | 26 | Fever, irritability, rash, HAD(6) |  |
| Jones M. (40) | CA 37+2wks | Irritability, lethargy, abnormal cry, reduced oral intake, hypothermia, respiratory difficulty, desaturation, seizure | GA 28+5weeks  B-MRI: extensive white matter and fronto-parieto-occipital cortical restricted diffusion |
| Berk et al. (41) | <28 | Clinical symptoms of sepsis without meningitis symptoms | GA 32+6wk, 2020g premature infant with NEC perforation |
| Midgley et al. (2) | Admitted to ICU  <28  (total 7pt) | Fever(4, 57%), rash(3, 43%), seizure(6, 86%), lethargy(1, 14%), irritability(5, 71%), difficulty breathing(3, 43%), rales(1, 14%), retraction(1, 14%), respiratory failure(3, 43%), tachycardia(6, 86%), cyanosis(2, 29%), mottling(2, 29%), abnormal heart sounds(1, 14%), hypotension(1, 14%), vomiting(2, 29%), watery stools(1, 14%), abdominal distension(1, 14%), jaundice(2, 29%), poor feeding(6, 86%), oxygen given(6, 86%), intubated(2, 29%) |  |
| Weichelt et al. (42) | 4-week-old | Pt1. Lethargy, diffuse hypotonia, apnea  Within 48hr, spasticity, hypertonia, status epilepticus, subclinical seizure  Pt2. Less severe compared Pt1  24hour, severe hypotonia, frequent apnea, Hypertonia, status epilepticus  Pt3. Progressive hypotonia, apnea, spasticity, epileptogenicity on EEG | GA 33.5 weeks triplet  Leukopenia, mildly elevated immature granulocytes  36mon follow up  Pt1 – Lt sided cerebral palsy, Cortical vision impairment, development delays  Pt2 – Cortical vision impairment, spasticity, developmental delays  Pt3.- progressed neurotypically through the first 3 years of development |
| Kadambari et al. (43) | <28 | Irritability, poor feeding, respiratory distress, signs of shock | 12mon follow up: well |
| Kirkley et al. (44) | 15 | Fussiness, grunting, distended abdomen, Fever, tachycardia  Marked abdominal distension, anorexia, fever, fussiness, irritability, poor feeding, grunting respiration | Abd X-ray: marked dilation of the ascending and transverse colon |
| Chamings et al. (45) | 23 | Sepsis-like |  |
|  | 12 | Sepsis-like |  |
|  | 17 | Sepsis-like, Meningitis |  |
|  | 10 | Sepsis-like |  |
|  | 9 | No info provided |  |
|  | 23 | Fever, diarrhea, tachycardia |  |
|  | 27 | No info provided |  |
|  | 20 | Fever, diarrhoea |  |
| Piralla et al. (46) | 15 | Irritability, fever, fussing, persistent crying, difficulty in feeding, axial hypotonia | Negative cranial ultrasound |
| Ancora et al. (47) | <31 | Fever, poor feeding, irritability, seizure, rash, pericardial effusion, respiratory insufficiency, shock, meningoencephalitis, diarrhea | 6 cases |
| Salavati et al. (48) | At birth | Hypotonic, hypokinetic, respiratory insufficiency, subclinical seizure | 32+4weeks, 2347g  feces HPeV test followed maternal history |
| Mei Jy Lim et al. (10) | At birth | Tachycardia, irritable, difficult to settle, Fever, desaturation, bradycardia, abnormal limb movements, seizure | Congenital  GA 36+4weeks, 3010g |
| Bucci et al. (49) | <30  (n=30) | Fever(100%), irritability(36.7%), poor feeding(23.3%), complaining behavior(23.3%), diarrhea(6.7%), rhinitis(6.7%), rush(3.3%), marbled skin(3.3%), exhaustible crying(3.3%), Nuchal stiffness(3.3%), Hyporeactivity(3.3%), Length of hospital stay(10.41±5.21) |  |
| Tierradentro-Garcia et al. (50) | 15 | Rash, poor feeding, irritability, fever, seizure, seizures | B-MRI both cerebral hemispheres asymmetrically mainly involving the deep white matter, periventricular WM, parietal and temporal lobes |
|  | 11 | Fever, irritability, apnea, right-sided clonic seizure | B-MRI bilateral relatively symmetric restricted diffusion of the subcortical and periventricular white matter |
| Hilbig et al. (51) | At birth | Petechiae, hypotonia, leukopenia, respiratory failure, seizure | Maternal fever, fetal tachycardia  GA 38+3wks, 4198g  Vertical transmission |
| Fox et al. (52) | 6 | Jaundice, decreased oral intake, febrile episode, Seizure, bradycardia, desaturation, rash | B-USG: subcortical white matter hypoechogenicity of both cerebral hemisphere, corpus callosum, temporal lobes |
|  | 16 | Decreased oral intake, irritability, fever | B-MRI: acute leukoencephalitis |
| Bozzola et al. (53) | <30  (n=8) | Fever, irritability, lethargy, poor feeding, diarrhea, rash, apnea/cyanosis, seizure, MRI abnormal | EEG: 7/8 patients had abnormal |
| Suthar et al. (13) | 13 | Seizure | GA 37+2weeks  B-MRI restricted diffusion and T2 FLAIR signal abnormality |
| DeArias et al. (54) | 14 | Fussiness, rash, decreased feeding, respiratory distress, seizure |  |
| Aizawa et al. (17) | <7 | Pt1. Fever, respiratory difficulty with nasal flaring, retraction, rash  Pt2. Rash, fever, abdominal distension  Pt3. |  |
| Turrubiates-Hernandez et al. (55) | 9 | Twin  Week sucking, apneas, irritable, pale, jaundice, generalized hypotonicity  Macular rash, pancytopenia, negative CRP  Vasopressor, nCPAP 🡪 IVIG | GA 36wks, RDS treatment  B-MRI: multiple hyperintensities in the frontal, parietal, temporal subcortical white matter |

HAD; hospital admission day, AOM; acute otitis media, CSF; cerebrospinal fluid, B-USG; brain ultrasonogram, B-MRI; Brain magnetic resonance image, ARI; acute respiratory insufficiency, EEG; Electroencephalogram, GA; gestational age, NEC; necrotizing enterocolitis, ICU; intensive care unit, Abd; abdomen, Pt; patient, HPeV; human parechovirus, FLAIR; fluid attenuated inversion recovery, CRP; C-reactive protein, nCPAP; nasal continuous positive airway pressure, IVIG; intravenous immunoglobulin, RDS; respiratory distress of newborn
